# Small molecules fail to induce direct reprogramming of adult rat olfactory ensheathing glia to mature neurons

**DOI:** 10.3389/fnmol.2023.1110356

**Published:** 2023-02-24

**Authors:** María Portela-Lomba, Diana Simón, David Fernández de Sevilla, Mª Teresa Moreno-Flores, Javier Sierra

**Affiliations:** ^1^School of Experimental Sciences, Universidad Francisco de Vitoria, Pozuelo de Alarcón, Madrid, Spain; ^2^Department of Anatomy, Histology and Neuroscience, School of Medicine, Universidad Autónoma de Madrid, Madrid, Spain; ^3^School of Medicine, Universidad Francisco de Vitoria, Madrid, Spain

**Keywords:** direct reprogramming, small molecules, neuro-regeneration, olfactory ensheathing glia, CNS injury

## Abstract

An approach to generate new neurons after central nervous system injury or disease is direct reprogramming of the individual's own somatic cells into differentiated neurons. This can be achieved either by transduction of viral vectors that express neurogenic transcription factors and/or through induction with small molecules, avoiding introducing foreign genetic material in target cells. In this work, we propose olfactory ensheathing glia (OEG) as a candidate for direct reprogramming to neurons with small molecules due to its well-characterized neuro-regenerative capacity. After screening different combinations of small molecules in different culture conditions, only partial reprogramming was achieved: induced cells expressed neuronal markers but lacked the ability of firing action potentials. Our work demonstrates that direct conversion of adult olfactory ensheathing glia to mature, functional neurons cannot be induced only with pharmacological tools.

## Introduction

Due to the low regenerative capacity of central nervous system (CNS), at present there is not an effective treatment after CNS injury or disease. An approach to generate new neurons in such situations is direct reprogramming: to convert somatic cells into neurons without going through a stem cell stage (Drouin-Ouellet et al., 2017; Gascón et al., 2017; Aydin and Mazzoni, 2019; Vignoles et al., 2019; Vasan et al., 2021; Bocchi et al., 2022). Fibroblasts (Vierbuchen et al., 2010), pericytes (Karow et al., 2018), and glial cells such as astrocytes (Heinrich et al., 2010; Masserdotti et al., 2015), microglia (Matsuda et al., 2019) or NG2 glia (Pereira et al., 2017) have been reprogrammed into functional neurons *in vitro* by transduction of viral vectors that express neurogenic transcription factors—NEUROD1 (Guo et al., 2014; Brulet et al., 2017), ASCL1 (Chanda et al., 2014; Liu et al., 2015), and Neurogenin 2 (NGN2) (Chouchane et al., 2017). Neurogenic transcription factors have been used on their own or in combination with other factors that provide a permissive epigenetic and metabolic context (Wapinski et al., 2013; Gascón et al., 2016) to enhance cell maturation, efficiency or specification (Masserdotti et al., 2015; Bocchi et al., 2022).

An alternative approach to convert astrocytes or fibroblasts into neurons is the use of small molecules that target neurogenic signaling pathways (Hu et al., 2015; Li et al., 2015; Zhang et al., 2015; Gao et al., 2017; Yang et al., 2019; Yin et al., 2019). Recently, *in vivo* chemical reprogramming of astrocytes in the adult mouse brain has been reported (Ma et al., 2021). Mechanistically, chemical reprogramming of astrocytes into neurons is mediated through both epigenetic and transcriptional regulation, activating proneural transcription factors such as NEUROD1, NGN2, and ASCL1 (Yin et al., 2019). Combinations of the following small molecules are commonly used in direct reprogramming strategies (Vasan et al., 2021; Bocchi et al., 2022; Wang et al., 2022): CHIR99021 (GSK3 inhibitor), SB431542 (TGFβ receptor inhibitor), DAPT (gamma-secretase inhibitor), forskolin (FSK) (PKA activator in cAMP signaling pathway), smoothened agonist (SAG) (Sonic hedgehog—SHH—activator), purmorphamine (SHH activator), valproic acid (HDAC inhibitor), IBET-151 (BET bromodomain inhibitor), isoxazole9 (ISX-9, inducer of adult neural stem cell differentiation), and/or LDN193189 (BMP pathway inhibitor).

Keeping in mind that the aim is to achieve neural repair after CNS injury or disease through induced neuron transplantation, the choice of the cell type to be reprogrammed is an essential aspect to consider. Thus, we selected olfactory ensheathing glia (OEG) as a candidate for direct reprogramming to neurons. An advantage of OEG over other cell types already reprogrammed to neurons, such as astrocytes or fibroblasts, is the reported capacity of OEG in promoting CNS regeneration (reviewed in Gómez et al., 2018). OEG is located in the mammalian olfactory system and provides a pro-regenerative environment for olfactory sensory neuronal axons (Gómez et al., 2018). In adult lifetime, olfactory sensory neurons are constantly renewed and OEG is responsible for facilitating axonal growth from the neuroepithelium to its targets in the olfactory bulb—mitral and tufted cells (Roet and Verhaagen, 2014; Gómez et al., 2018). OEG neuro-regenerative capacity has been tested in an *in vitro* model of axotomized rat retinal ganglion neurons (RGNs): OEG olfactory bulb and mucosa—derived but not lung or skin fibroblasts mediate *in vitro* axonal regeneration of RGNs (Moreno-Flores et al., 2003; García-Escudero et al., 2012; Portela-Lomba et al., 2020). OEG neuro-regenerative capacity has also been assessed *in vivo* in rat models of spinal cord injury (SCI) (Moreno-Flores et al., 2006; Khankan et al., 2016; Thornton et al., 2018) and its reparative ability is due to a combination of neurotrophic and neuroprotective factors (reviewed in Gómez et al., 2018).

If we add to the neuro-regenerative capacity of OEG cultures, the conversion to neurons of a fraction of their population through reprogramming techniques, the engraftment of OEG and OEG induced neurons (OEG-iNs) could enhance neural repair at the damaged site. Recently, it was reported that OEG from adult mice could be directly reprogrammed into neuronal cells by the single transcription factor NGN2 (Sun et al., 2019). In the present work, we assess the feasibility of directly converting OEG into neurons by means of small molecule induction, overcoming the concern of introducing foreign genetic material to cell cultures that might be potentially used for cell-based therapy. We characterized a primary OEG culture from olfactory bulb of adult rats (OEG-ROB) that showed neuro-regenerative properties and, after chemical induction, exhibited morphological and immunolabeling neuronal-like features but were not electrophysiologically competent. Our work reveals that direct conversion of adult OEG to mature, functional neurons cannot be induced only with pharmacological tools.

## Materials and methods

### Animals

Adult male and pregnant female Wistar rats (RccHan^®^:WIST) were obtained from Envigo (Envigo RMS Spain, SL) and sacrificed upon arrival. All animal experimentation was carried out in animal facilities of Universidad Francisco de Vitoria, complying with the European Council Directive 2010/63/UE and approved by national and institutional bioethics committees. Animals were maintained on a 12-h light/12-h dark cycle in a day and were supplied with regular food and water *ad libitum*.

### ihOEG cell cultures

Immortalized human OEG (ihOEG) cell lines Ts14 and Ts12 were maintained as previously described (García-Escudero et al., 2011; Plaza et al., 2016). Cells were grown in ME medium [DMEM/F12 (Gibco) supplemented with 10% FBS (GE Healthcare Hyclone), 2 mM glutamine (Lonza), 20 μg/mL pituitary extract (Gibco), 2 μM forskolin (FSK; Sigma), and antibiotics (P/S/A, penicilin/estreptomicin/anfotericin; Lonza)] and maintained at 37°C in 5% CO_2_.

### Rat OEG primary culture

Olfactory ensheathing glia from rat olfactory bulbs (OEG-ROB) was obtained as follows. Olfactoy bulbs from p21; RccHan^®^: WIST rats were dissected and, after removal of meninges, the external layer was separated and cut with a scalpel blade until <1 mm pieces were obtained. Then, the pieces were placed in a 15 mL Falcon, the supernatant was removed and 2 mL of 0.1% tripsin was added to the pellet. They were incubated for 15 min at 37°C with intermittent shaking. After adding 4 mL of HBSS+ (HBSS supplemented with 20% fetal bovine serum (FBS; Gibco) and 2,000 U/mL DNAse) the suspension was centrifuged at 200 × g for 5 min. The pellet was resuspended in 2 mL of HBSS+ and it was mechanically disaggregated by pipetting 10 times with a glass Pasteur and other 10 times with a pre-squeezed glass Pasteur. The suspension was centrifuged at 200 × g for 10 min. Subsequently, the supernatant was aspirated, cells were resuspended in 3 mL of ME media and distribuited in 3 p60 culture plates (1 mL per p60; Falcon), pre-treated with poly-L-lysine (PLL, 10 μg/mL; Sigma).

### Rat neuron primary culture

Cerebral cortex from E18 RccHan^®^: WIST rats was dissected, meninges were removed and transferred to a papain plate, where they were cut with a scalpel blade until <1 mm pieces were obtained. They were incubated for 30 min at 37°C with intermittent shaking. Then, they were mechanically disaggregated by pipetting with a glass Pasteur 10–15 times until a homogeneous suspension was obtained. The suspension was passed through a 0.75 mm mesh to remove clumps and centrifuged for 5 min at 200 × g. Subsequently, the supernatant was aspirated, cells were resuspended in 5 mL of NB-B27 Plus medium [Neurobasal Plus (NB, Gibco) supplemented with B27 Plus (Gibco), 2 mM glutamine (Lonza) and P/S/A (Lonza)] and 50,000 cells were plated on glass coverslips (12 mm, ThermoScientific), pre-treated with PLL-Laminin (10–5 μg/mL).

### Astrocyte primary culture

Astrocytes were isolated from cortex of postnatal 0–2 days old (P0-2) C57BL6 mice. Briefly, cerebral cortex was dissected and, after removal of meninges, cut with a scalpel blade until <1 mm pieces were obtained. They were passed five times through a 5 mL pipette and incubated for 30 min at 37°C with intermittent shaking. Then, they were mechanically disaggregated by pipetting with a glass Pasteur 20–25 times until a homogeneous suspension was obtained. The suspension was then passed through a 0.75 mm mesh to remove the remained aggregates and centrifuged for 5 min at 200 × g. Subsequently, the supernatant was aspirated, cells were resuspended in the corresponding volume of M10 (1 mL per 4 hemispheres) and 1 mL of the supernatant was plated in a T75 culture flask (hereafter T75 flask; Falcon), pre-treated with poly-L-lysine (PLL, 10 μg/mL; Sigma). After 7–10 days, when the cells reached 90–95% confluence, the T75 flasks were shaken O/N at 37°C to obtain a purified astrocyte culture.

### OEG-ROB co-cultures with retinal ganglion neurons

OEG-ROB-RGNs co-cultures were carried out to assess the neurogenerative capacity of OEG-ROB as previously described (Portela-Lomba et al., 2020). In brief, OEG-ROB cells were plated on 12 mm coverslips in a 24-well plate (M24; Cultek), with the aim of forming a monolayer the following day. RGNs were extracted from retinas of adult rats (Wistar rats; Envigo) by sectioning the optic nerves and were dissociated using a papain kit (Worthington Biochemical Corporation). After 96 h, co-cultures were fixed for immunostaining.These co-cultures are an adult axonal regeneration *in vitro* model because adult neurons are extracted after cutting optical nerves and these neurons lose their axons in the process.

To assess the regenerative capacity of OEG-ROB, RGNs grown in co-culture were immunolabeled with SMI31 [phosphoepitope in microtubule associated protein (MAP) 1B and neurofilament H axonal markers] and 514 (against MAP2A,B, somato-dendritic marker) and analyzed with the 40X objective of an inverted fluorescent microscope (DMi8, Leica). At least 30 fields were randomly imaged and at least 200 neurons were quantified in each preparation. Experiments were repeated independently at least three times.

Immunofluorescence signals were quantified with ImageJ software (ImageJ; NIH) and axon length was measured using the NeuronJ plugin. Quantification of axonal regeneration was determined by calculating: (1) the percentage of neurons with axons, detected with SMI31, vs. the total number of neurons, labeled with 514; (2) the mean axon length per neuron, by calculating the ratio of the lengths (μm) of all axons out of the total number of counted neurons.

### OEG-ROB reprogramming with small molecules

M24 cell plates (IBIDI) were treated with polyornitine (20 μg/mL)–laminin (5 μg/mL) before 15,000 cells/well were plated in ME medium. After 24 h, 60–70% confluence was reached and different combinations and concentrations of the following small molecules were added to each well (see Supplementary Table 1), in differentiation medium [1:1 DMEM/F12:NB (Gibco), glutamax (Gibco), P/S/A (Lonza), glucose 3,5 mM (Sigma), N2 (Gibco) and B27 (Gibco), plus BDNF (brain derived neurotrophic factor), GDNF (glia derived neurotrophic factor), and NT3 (neurotrophin 3) at a final concentration of 20 ng/mL]: CHIR99021 (SigmaSML1046), SB431542 (SigmaS4317), DAPT (SigmaD5942), LDN193189 (SigmaSML0559), ISX-9 (Tocris4439), Forskolin (SigmaF6886), SAG (SigmaSML1314), Purmorphamine (SigmaSML0868), IBET-151 (SigmaSML0666), Valproic acid (SigmaPHR1061), DMSO (ThermoFisher 10103483). Induction medium (small molecules + differentiation medium) was refreshed every 4 days. Depending on the trial, small molecules were either maintained until the end of the assay or removed at some point in the reprogramming process.

### Generation of OEG-ROB-GFP cell line

OEG-ROB-GFP cell line was generated by infecting OEG-ROB with E-GFP encoding lentivirus. Cells were infected and incubated with a multiplicity of infection (MOI) of 10 infectious units/cell overnight (O/N) in M10 medium. Next day, cells were washed and maintained for 48 h in M10, for transgene expression. OEG-ROB cells exhibiting high E-GFP expression were selected and sorted by flow cytometry (MoFlo Astrios Sorter; Beckman Coulter) and named as OEG-ROB-GFP. OEG-ROB-GFP cell line was tested for the maintenance of its neuro-regenerative capacity *in vitro* in the OEG-RGN co-culture model previously described (data not shown).

### OEG-ROB induced neurons co-culture with astrocytes

Aiming to improve reprogramming conditions, we co-cultured OEG-ROB-iNs with mouse postnatal astrocytes. A monolayer of mouse postnatal astrocytes was obtained by plating 75,000 cells in M24-I plates (IBIDI) pretreated with polyornitine (20 μg/mL)–laminin (5 μg/mL). The remaining astrocytes were plated in 150 mm plates (P150), pretreated with PLL to get conditioned differentiation medium from the astrocytes. After 48 h, 25,000 OEG-ROB-GFP were plated onto the monolayer. Next day, the medium was changed to conditioned medium containing the different combination of small molecules (conditioned induction medium: small molecules + conditioned differentiation medium). The following combinations of small molecules were assessed: FSK 20 μM + CHIR 1.5 μM // FSK 10 μM + CHIR 1.5 μM // FSK 20 μM + SAG, Purmorphamine 0.4 μM // DMSO. Conditioned induction medium was refreshed every 4 days. The co-culture was fixed after 60 days.

### Immunofluorescence

Cells were fixed with 4% PFA, permeabilized and blocked with PBS-TS (PBS, 0.1% Triton, 5% FBS) for 30 min at RT and then incubated with the corresponding primary antibody diluted in PBS-TS at 4°C O/N. Next day, they were washed 3 times with 1X PBS for 5 min and incubated with the corresponding fluorescent secondary antibody (Alexa), diluted in PBS-TS, for 1 h at RT in the dark. Three 5-min washes were performed with 1X PBS and were additionally incubated for 5 min with DAPI (4′6-Diamidino-2-phenylindole; 1 μg/mL, MERCK). Samples were mounted with fluoromount (Southern Biotech) and observed under an inverted fluorescent microscope (DMi8, Leica). Antibodies and antibodies concentrations are listed in Supplementary Table 2. For each condition, fields were photographed at random and the intensity density of the somas was quantified in an automated and blind way, taking as region of interest the DAPI labeling. Immunofluorescence signals were quantified using ImageJ software (ImageJ; NIH).

### Electrophysiology

After 30 days post induction with small molecules, cells were recorded using the patch-clamp technique in their whole-cell configuration in voltage-clamp and current-clamp modes. Recordings were made with borosilicate pipettes (OD-ID: 1.5–0.86; Sutter Instrument CO, Novato, CA), with a resistance of 4–8 MΩ and were filled with an intracellular solution (5 mM KCl, 20 mM HEPES, 2 mM CaCl2, 2 mM MgCl2, 0.6 mM EGTA, 130 mM K-gluconate, 2 mM ATP, 0.2 mM GTP, 20 mM phosphocreatine, and 50 U/mL creatine phosphokinase; pH 7.3 and osmolarity 286 mOsm). Recordings were performed at a temperature of 30°C with a constant flow of 2 mL/min extracellular solution (150 mM NaCl, 2.5 mM KCl, 10 mM HEPES, 30 mM glucose, 1 mM MgCl2, and 2 mM CaCl2; pH 7.3). Action potentials and sodium currents were evoked by depolarizing pulses of current and voltage. Cells were accepted only when the seal resistance was above 1 GΩ and the series resistance did not change by 20 % during the experiment. Signals were filtered at 3 KHz and sampled at 10 KHz with a Digidata 1500A analog-to-digital conversion card (Molecular Devices, Sunnyvale, CA). Rat embryonic cortex neurons cultured *in vitro* between 7 and 14 days were recorded as positive control. After obtaining a stable recording of action potentials and sodium currents, recordings were blocked by adding tetrodotoxin (TTX; 0.5 μM, Abcam), a blocker of voltage-dependent sodium channels, to the extracellular solution.

### Statistical analysis

For statistical comparisons of the regenerative capacity study, two-factor analysis of variance (One-way ANOVA) was performed, followed by Tuckey's *post-hoc* test for multiple comparison between means. Statistical significance was established at a value of *p* < 0.05.

## Results

### Adult rat olfactory bulb ensheathing glia shows neural regenerative properties

**Figure 1:**
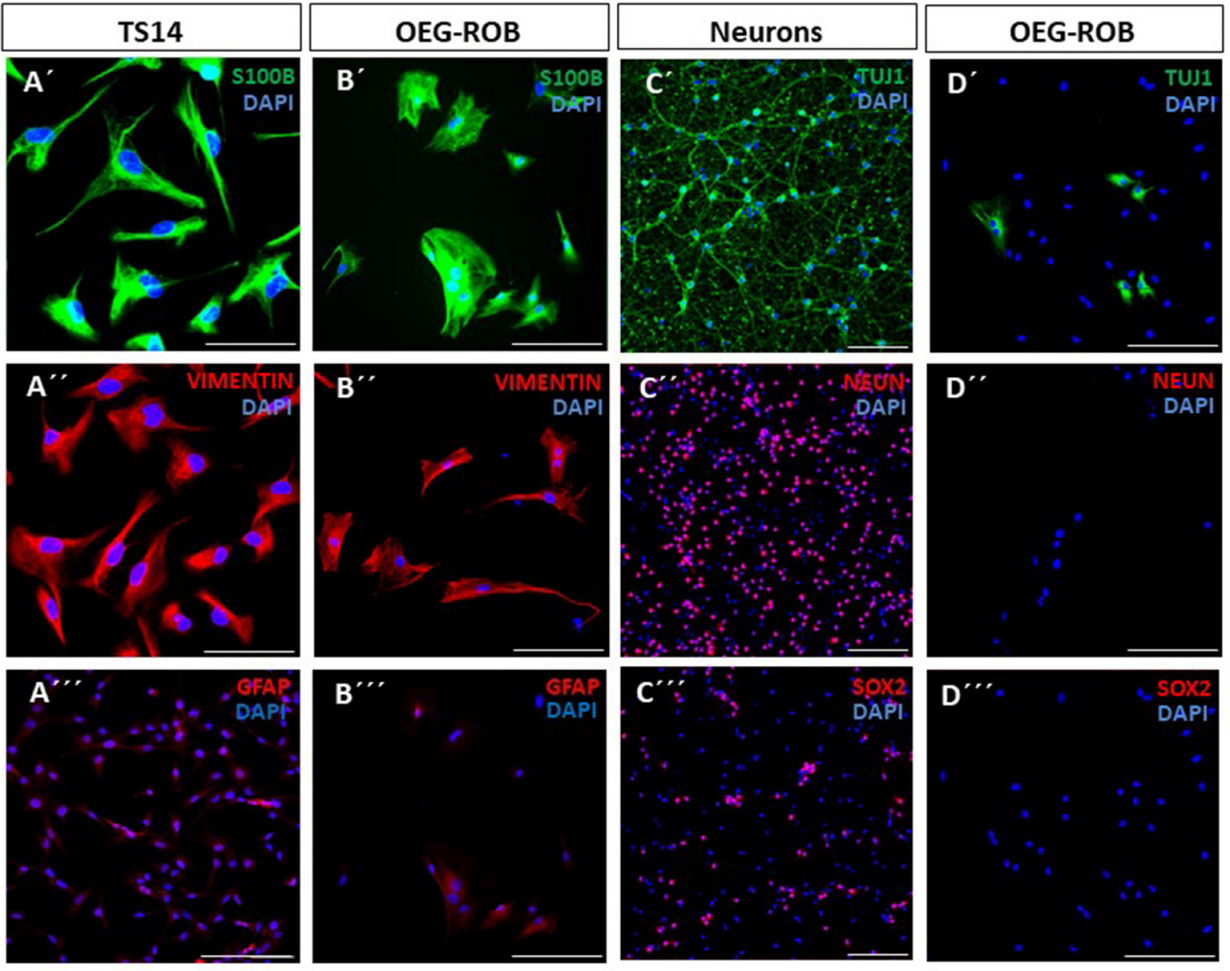
Characterization of OEG-ROB cells. Expression analysis of glial and neuronal markers was performed by immunofluorescence in primary cultures of rat olfactory bulb ensheathing glia (OEG-ROB); TS14 immortalized OEG line and a neuronal primary culture from rat embryonic cortex (E18) were used as positive controls. Glial markers S100β (green) and vimentin (red) were expressed in TS14 **(A′, A″)** and OEG-ROB **(B′, B″)**, while GFAP (red) was also expressed in both OEG cell types **(A^‴^, B^‴^)** but with a more diffused pattern. Proneural gene NeuN expression (red) was absent from OEG-ROB, while neuronal culture stained positively for this marker **(C″, D″)**. However, Tuj1 (green) not only stained embryonic neurons, but was also detected in OEG-ROB **(C′, D′)**. OEG-ROB population was not contaminated with neural precursors as SOX2 positive staining (red) was not detected in the OEG-ROB culture, while neural progenitor cells from rat embryonic cortex expressed this neural precursor marker **(C^‴^, D^‴^)**. Nuclei were stained with DAPI (blue). For quantitative data, see Supplementary Figure 1. Scale bar: 100 μm.

OEG primary cultures were prepared from rat olfactory bulbs (P-21), OEG-ROB from now on. We carried out an expression analysis of OEG markers -S100β (S100 glial calcium binding protein B), GFAP (glial fibrillary acid protein) and vimentin—by immunofluorescence techniques. As positive controls we used TS14 cell line—an immortalized OEG cell line previously described in García-Escudero et al. (2011) and Plaza et al. (2016)—and a primary culture of neurons. OEG-ROB cultures were positive for S100β (99 ± 0.5%) and vimentin (98.5 ± 4.2%), exhibiting a diffused staining pattern for GFAP, as observed in TS14 (Figures 1A′–B^‴^; Supplementary Figure 1A). We then verified that OEG-ROB cells did not express neuronal markers by labeling β3-tubulin (Tuj1) and neuronal nuclear antigen (NeuN). NeuN expression is absent from OEG-ROB, but Tuj1 immunoreactivity was higher than expected (30.6 ± 18%) (Figures 1C′–D″; Supplementary Figure 1B). To rule out the presence of neuronal precursors we performed immunofluorescence to detect SOX2, expressed in neural progenitor cells; we used a primary culture from rat embryonic cortex (E18) as a positive control, which due to its embryonic stage is a niche for neuronal precursors. Our results exclude the presence of neuronal precursors in our OEG-ROB cell line: the embryonic cortex culture contains 34.5 ± 3.5% of SOX2-positive neuronal precursors but OEG-ROB cells do not express SOX2 (Figures 1C^‴^, D^‴^; Supplementary Figure 1B).

**Figure 2:**
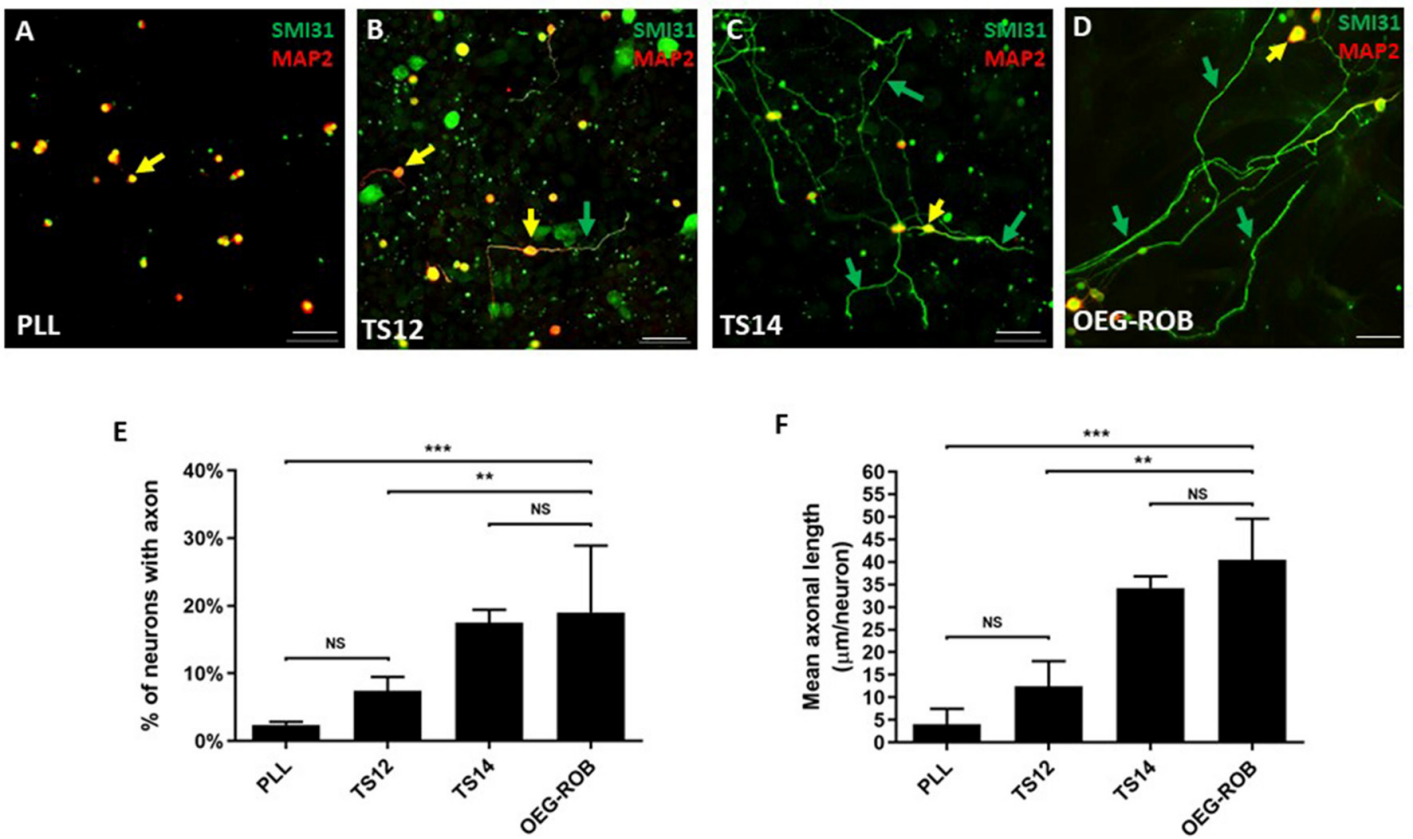
Analysis of the regenerative capacity of OEG-ROB in an *in vitro* model of adult axonal regeneration. **(A–D)** show representative images of axotomized retinal ganglion neurons after 96 h in co-culture with OEG. OEG human cell line TS14 was used as a positive control for neuroregeneration **(C)** and human cell line TS12 **(B)** and PLL **(A)** correspond to low regeneration and negative controls, respectively; **(D)** corresponds to OEG-ROB. Green arrows indicate axons positive for axonal marker SMI31 (green) and yellow arrows indicate somas and dendrites positive for somatodendritic marker MAP2 (red) (antibody 514 against MAP2A&B). Histograms represent the mean ± SD of the quantifications: percentage of retinal neurons extending an axon **(E)** and the mean axon size expressed as μm/neuron **(F)**. Statistical tests applied were one-way ANOVA and *post-hoc* Tukey test (***p ≤ 0.001; **p ≤ 0.01; NS, not significant) for multiple comparisons between means (*n* = 4, per experiment ≥ 30 fields were analyzed). Scale bar: 50 μm.

Next, we assessed the regenerative properties of OEG-ROB. For this purpose, we conducted an *in vitro* regeneration assay, co-culturing OEG-ROB with axotomized adult rat retinal ganglion neurons (RGNs) (see Section Materials and methods). TS14, a line with regenerative capacity, and TS12, an immortalized OEG line with low regenerative capacity, were used as controls (García-Escudero et al., 2011; Plaza et al., 2016) and we verified that axotomized RGN did not inherently extend axons growing RGNs on poly-L-L-lysine (PLL). Axonal regeneration was quantified using immunostaining against SMI31 as an axonal marker and against MAP2A/B to identify the somatodendritic compartment. Two parameters were assessed to determine regenerative capacity: the percentage of RGNs that extended axons and the mean axonal length/neuron of these axons. After analysis of these parameters, we observed that the regenerative capacity of OEG-ROB is similar to that of our positive control. Percentage of neurons with axons is 19.6 ± 3.6% and the average axonal length is 40.5 ± 9.1 micrometers/neuron (Figure 2). Moreover, a statistically significant higher regenerative capacity of both parameters was observed with respect to the two negative controls (Figure 2).

Overall, the OEG-ROB line expresses OEG markers but does not express neuronal or neuronal precursor markers. Furthermore, these cells show pro-regenerative properties in an *in vitro* model of adult axonal regeneration.

### OEG-ROB acquires neuronal characteristics after treatment with small molecules but these fail to induce a mature and functional neuronal phenotype

**Figure 3:**
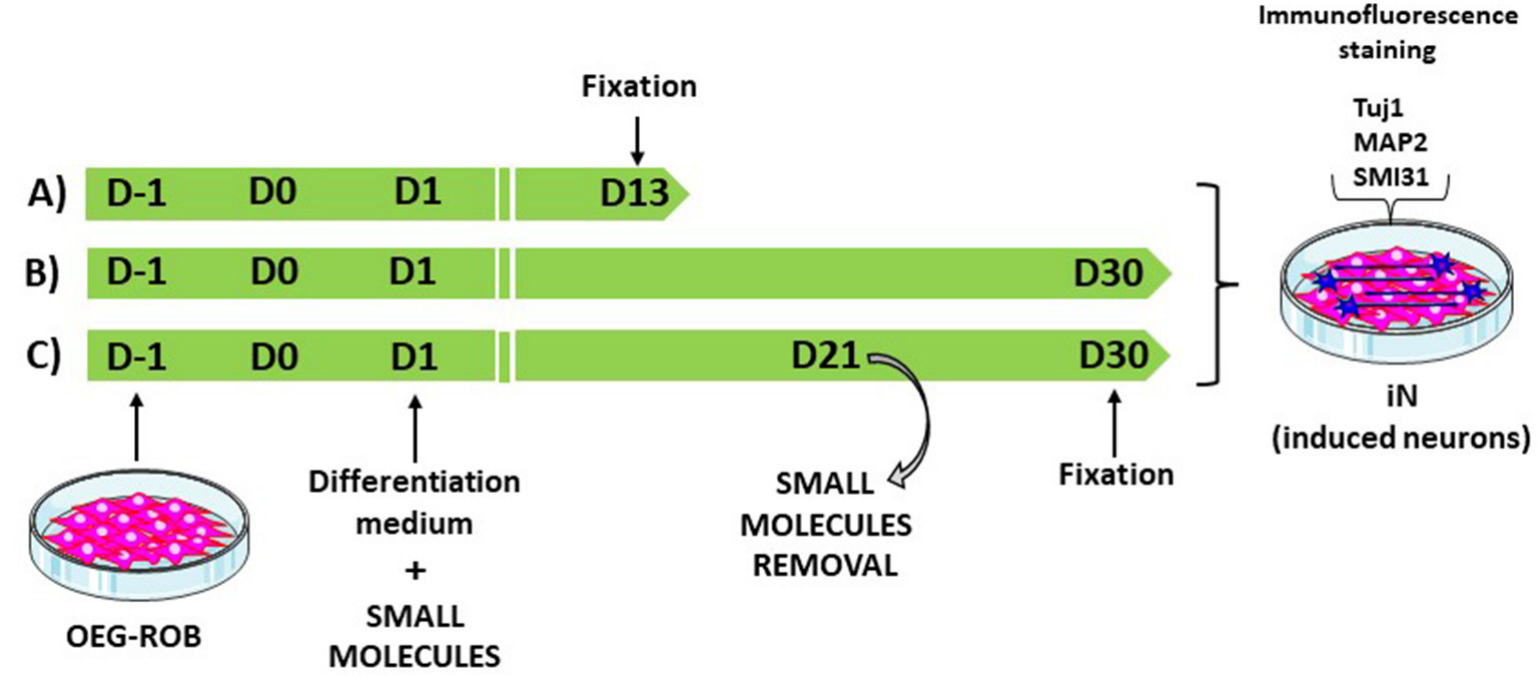
OEG-ROB reprogramming workflow. To induce reprogramming, small molecules were added to the differentiation medium from the first day of induction (D1) and were maintained until the end of the trial, 13 days (D13) or 30 days (D30) post induction **(A, B)**. In **(C)**, small molecules were removed at day 21 (D21) of the reprogramming process and OEG-ROB were left in differentiation medium for further maturation until day 30 (D30). Neuronal induction was assessed *via* expression of neuronal markers Tuj1, MAP2, and SMI31.

**Figure 4:**
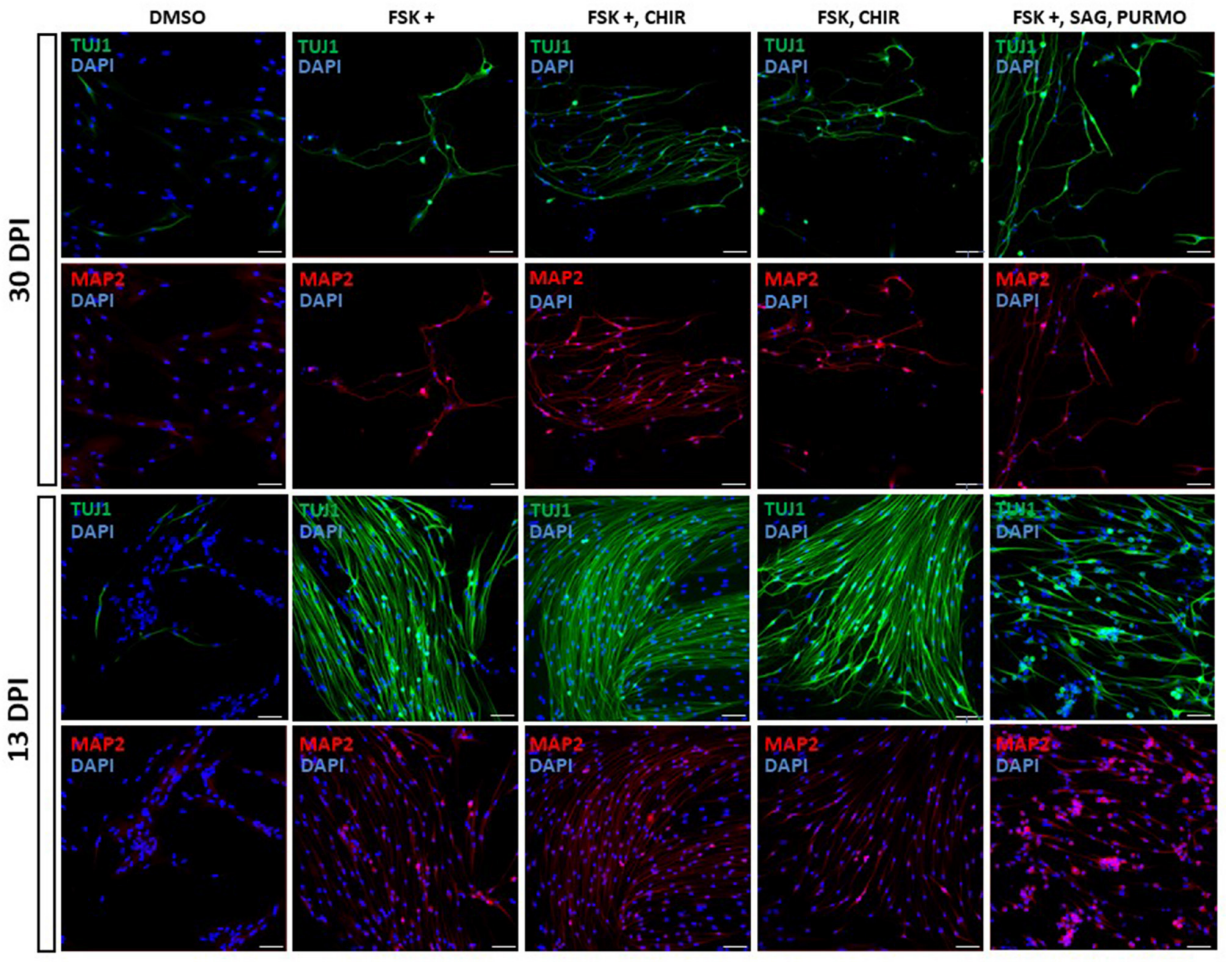
Proneural genes expression in reprogrammed OEG-ROB cells. Expression analysis of neuronal markers was carried out by immunofluorescence after 13 and 30 days of induction with small molecules (see legend for cocktails and concentrations). In both conditions, OEG-ROB cells showed positive staining for Tuj1 (green) and for MAP2 (red) indicating a shift to neuronal fate. Cell number was compromised after 30 days of induction with small molecules but surviving cells changed their morphology, showing compact cell bodies and neurite-like structures. When time of induction was reduced to 13 days, cell viability increases and reprogrammed OEG-ROB cells aggregate into bundles. Nuclei were stained with DAPI (blue). For quantitative data, see Supplementary Figure 1. DPI, days post-induction; DMSO, DMSO 0.1%; FSK, forskolin 10 μM; FSK+, forskolin 20 μM; CHIR, CHIR99021 1.5 μM; SAG, smoothened agonist 0.2 μM; Purmo, purmorphamine 0.2 μM; Scale bar: 50 μm.

We screened small molecules frequently used in neuronal reprogramming or differentiation (Li et al., 2015; Zhang et al., 2015; Gao et al., 2017; Yang et al., 2019; Yin et al., 2019): forskolin, CHIR99021, SAG, purmorphamine, ISX9, SB431542, DAPT, LDN 193189, IBET-151 and valproic acid. To induce reprogramming, small molecules were added to the differentiation medium from the first day of induction and were maintained until the end of the trial or removed at some point in the reprogramming process (Figure 3; Supplementary Table 1). As a result of the screening, the following set and concentrations were selected: forskolin 20 μM; forskolin 20 μM and CHIR 1.5 μM; forskolin 20 μM, SAG 0.2 μM and purmorphamine 0.2 μM; forskolin 10 μM and CHIR 1.5 μM. We then kept OEG-ROB in presence of these small molecules for 30 days and monitored expression of proneural markers Tuj1 and MAP2 by immunofluorescence. Under these conditions, a decrease in cell number was observed but surviving cells changed dramatically their morphology, showing compact cell bodies and neurite-like structures in addition to positive staining for Tuj1 and MAP2 (Figures 3, 4; Supplementary Figures 1E, F). These data indicated a shift of OEG-ROB to neuronal fate after small-molecule treatment. Following induction, OEG-ROB will be referred to as OEG-ROB induced neurons (OEG-ROB-iNs).

**Figure 5:**
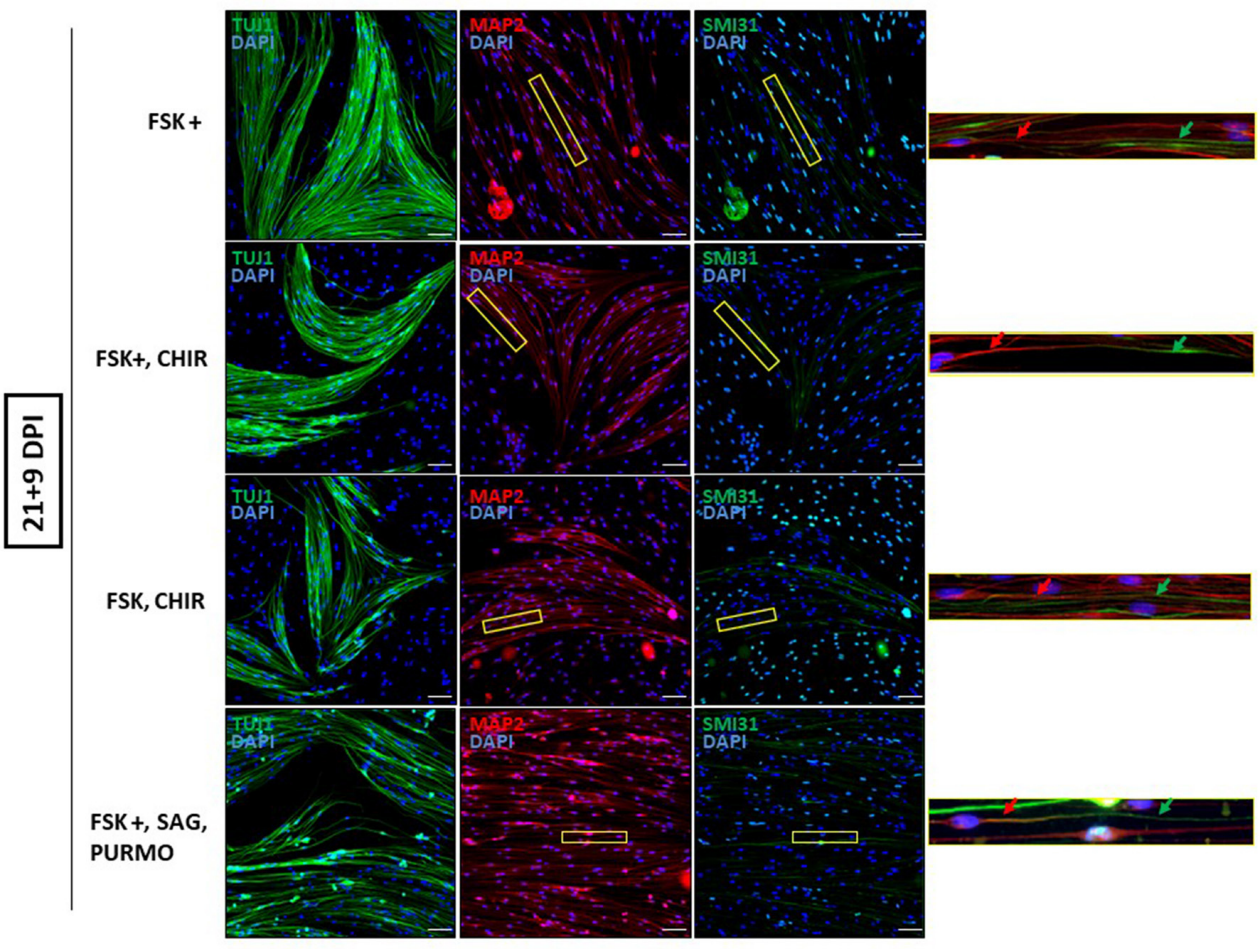
Mature neuronal marker expression in reprogrammed OEG-ROB cells. Removing the medium with small molecules at day 21 and adding differentiation medium until day 30, allowed an increase in cell survival together with the development of an elongated shape and cell aggregation into bundles. Reprogrammed OEG-ROB cells not only expressed proneural markers Tuj1 (green) and MAP2 (antibody 514 against MAP2A&B) (red) but also SMI31 in MAP1B and neurofilament H proteins (green), which is a component of mature axons and is characteristically expressed in a gradient manner, as can be observed in the figure enlargements: red arrows show MAP2 expression while green arrows highlight SMI31 expression more distally in the same cell axon. Nuclei were stained with DAPI (blue). DPI, days post-induction; FSK, forskolin 10 μM; FSK+, forskolin 20 μM; CHIR, CHIR99021 1.5 μM; SAG, smoothened agonist 0.2 μM; Purmo, purmorphamine 0.2 μM; Scale bar: 50 μm.

Next, we reduced the time OEG-ROB cells were in the presence of selected drugs to increase cell viability without compromising cell reprogramming. We did so either by fixing them at day 13 or by removing the medium with small molecules at day 21 and adding differentiation medium until day 30 (Supplementary Table 1; Figure 3). In both conditions we observed an increase in cell survival together with the development of an elongated shape and cell aggregation into bundles (Figures 4, 5). In addition, not only Tuj1 and MAP2 positive staining was maintained (Figures 4, 5; Supplementary Figures 1C, D) but, after 21 days of induction and small molecule retrieval, reprogrammed cells presented a more mature neuronal phenotype, evidenced by the expression of SMI31 in positive gradient from the soma toward the neurite tip, a pattern characteristic of mature axons (Bush et al., 1996) (Figure 5, magnifications).

To determine the functionality of OEG-ROB-iNs, we performed electrophysiological assays using the patch-clamp technique. Recordings were performed after 30 days post induction with small molecules and rat embryonic cortex neurons were used as a positive control. In these neurons, we recorded a sodium input of ~500 picoamperes (pA) that fired action potential trains and this sodium current was blocked by exposure to TTX, a neurotoxin that blocks voltage-dependent sodium receptors, revealing that the sodium current is specific to the voltage-dependent sodium channels characteristic of a neuron (Supplementary Figure 2). However, when OEG-ROB-iNs electrophysiological properties were assayed, neither sodium currents nor action potentials were recorded (Supplementary Figure 2).

We were aware that electrophysiological assays should be carried out on older OEG-ROB-iNs in long term culture, to demonstrate whether these cells are functional. However, after 30 days in the presence of small molecules, cell viability of reprogrammed cells was dramatically reduced. To overcome this issue, we tried a co-culture system: we established an OEG-ROB-GFP cell line *via* viral GFP transduction—to identify OEG-ROB-iNs—and co-cultured this line on a P0-2 mouse astrocyte layer in the presence of the previously selected set of small molecules. The co-culture was viable for 60 days, after which cells began to detach from the plates and were fixed. Few GFP positive cells were observed and, although the morphology resembled that of more mature neurons, especially in the conditions FSK 20 μM + CHIR 1.5 μM, GFP positive cells failed to express Tuj1 or Synapsin markers (Supplementary Figure 3). Expression of Tuj1 was observed in GFP negative cells, but this may be due to the effect of the small molecules on the astrocyte layer as reported in Zhang et al. (2015) and Gao et al. (2017).

Taken together, these results indicate that OEG-ROB can be reprogrammed with small molecules into a neuronal-like phenotype, with morphological and immunocytochemical neuronal characteristics, but they are not induced to mature and functional neurons.

## Discussion

A recent approach to foster CNS regeneration after injury is to generate new neurons in the damaged site *via* reprogramming somatic cells, without going through a stem cell stage (Bocchi et al., 2022). Compared to the generation and engrafting of induced pluripotent stem cells, this strategy has proven to overcome the barrier of tumorigenesis and indeterminate differentiation (Bellin et al., 2012). In the present work, we assess the feasibility of directly converting OEG into neurons by means of small molecule induction. Most of glia-to-neuron conversion research has been carried out using virus-mediated ectopic expression of transcription factors (Bocchi et al., 2022), having been recently reported that OEG from adult mice could be directly reprogrammed into neuronal cells by the single transcription factor NGN2 (Sun et al., 2019). Chemical induction overcomes the concern of introducing foreign genetic material to cell cultures that might be potentially used for cell-based therapy. Also, an advantage of OEG over other cell types is the reported capacity of OEG in promoting CNS regeneration, either *in vitro* or *in vivo* (reviewed in Gómez et al., 2018). In the model of OEG-retinal ganglion neurons (RGNs) co-culture described above, OEG olfactory bulb and mucosa -derived but not lung or skin fibroblasts mediate *in vitro* axonal regeneration of axotomized RGNs (García-Escudero et al., 2012). This pro-regenerative capacity supports the election of OEG as a candidate for direct reprogramming vs. other cell types. Furthermore, with an eye on its clinical application, mucosa OEG cells have the additional advantage that they can be easily obtained from the patient by a simple biopsy, so autologous therapies can be performed and avoid post-transplant rejections. Therefore, if we add to the neuro-regenerative capacity of OEG, the conversion to neurons of a fraction of their population through reprogramming techniques, the engraftment of OEG and OEG induced neurons could enhance neural repair at the damaged site.

To characterize our OEG-ROB line we analyzed the expression pattern of glial and neuronal markers and its neuro-regenerative capacity. As expected, similar expression of glial markers was observed in OEG-ROB when compared to TS14 positive control, with GFAP diffused labeling for both cell lines. Two types of OEG immunodetections in culture are described in the literature, one with intense and fibrous GFAP labeling and the other with weak and diffuse GFAP (Franceschini and Barnett, 1996). In our hands, both human (Lim et al., 2010) and rat (Moreno-Flores et al., 2003) OEG lines have shown diffuse GFAP immunostaining. Regarding expression of neuronal markers in OEG-ROB cells before induction, NeuN expression was absent from the glial cell line, but a small proportion of OEG-ROB cells labeled positive for Tuj1. However, previous studies have reported a diffuse cytoplasmic expression of Tuj1 in OEG lines (García-Escudero et al., 2012) and, likewise, a constitutive expression of Tuj1 not associated with cell passage has been described in human astrocyte cultures (Dráberová et al., 2008). Additionally, no SOX2-positive labeling was detected in the OEG-ROB cell cultures, ruling out the presence of residual neural precursors. After conducting co-culture assays of OEG-ROB cells with axotomized adult rat RGNs, OEG-ROB showed a significant regenerative capacity, by comparison to high and low neuro-regenerative OEG lines, in accordance with expected results (Plaza et al., 2016).

Based on an initial screening, we selected a set of small molecules known to modulate signaling pathways that play important roles in neural development, thus used together with the expression of transcription factors to increase reprogramming efficiency or inducing neural conversion on their own: forskolin, forskolin/CHIR99021 or forskolin/SAG/purmorphamine. Forskolin is a cAMP/PKA activator inducing cell differentiation and epithelial-mesenchymal transition that has been reported to facilitate chromatin accessibility to NGN2 (Smith et al., 2016). CHIR99021 is a GSK3 inhibitor, consequently activating WNT signaling pathway involved in neural development, adult neurogenesis and neuronal differentiation (Clevers, 2006); the combination of CHIR99021 with forskolin and other small molecules has been shown to promote the conversion of astrocytes (Yin et al., 2019) or fibroblasts (Yang et al., 2019) into neurons. SHH signaling pathway is enhanced by SAG (Smoothened agonist) and purmorphamine and regulates multiple aspects of animal development, tissue homeostasis and regeneration (Ingham, 2022). Activation of the SHH pathway has been reported to promote neuronal differentiation from human fetal astrocytes (Zhang et al., 2015) and purmorphamine was included in a cocktail that converted human fibroblasts into neurons (Yang et al., 2019).

Induction of OEG-ROB with our set of small molecules produced neuron-like cells with neurite-like extensions and were positively stained for proneural markers, as pharmacological modulation of signaling pathways could provide a permissive environment for the expression of neurogenic factors. The observed decrease in cell viability after 30 days induction is consistent with results from the group of Dr. Dai (Yang et al., 2019) who reported severe cell death after continuous exposure to small molecules. A possible explanation could be the metabolic change that occurs in cell reprogramming, inducing cellular oxidative stress and, consequently, causing cell death by ferroptosis (Gascón et al., 2016). Interestingly, a small percentage of cells from untreated groups was positive for Tuj1, indicating that the induction medium itself could activate Tuj1 expression. Thus, several markers as well as morphological features should be considered to characterize induced neurons. Small molecule retrieval after 21 days of induction and maintenance of induced cells in differentiation medium until day 30 not only preserved proneural gene expression and increased cell viability but allowed a more mature neuronal phenotype, evidenced by a gradient expression of SMI31. This is in accordance with previous results (Yang et al., 2019) who achieved a reprogramming peak at day 14 post induction, shown by Tuj1 immunostaining analysis. Unfortunately, induced cells did not produce inward sodium currents nor fired action potentials as expected from mature, functional neurons.

The fact that similar drug combinations have different reprogramming effects on different cell types has already been reported. For example, Zhang et al. (2015) could reprogram human astrocytes of fetal origin from the cortex or midbrain, to neurons, with a combination of small molecules, but not from spinal cord. Likewise, the same small molecule cocktail was not sufficient to reprogram mouse astrocytes, suggesting that different glial cell lineages may be sensitive to different sets of small molecules.

It seems plausible that after initial induction to neural fate, OEG-ROB cells require a maturation environment that could be provided by the interaction with a feeder cell layer. Indeed, it has been reported (Yang et al., 2019) that for long-term culture, iNs need to be plated on mouse postnatal astrocytes after treatment with small molecules. Therefore, we considered the option of establishing an OEG-ROB-GFP cell line *via* viral GFP transduction and to co-culture this line on an astrocyte layer. Since small molecules could affect astrocyte phenotype, OEG-ROB induced neurons would be identified by fluorescence. However, only a small number of GFP positive cells survived the established culture time (60 days post induction with small molecules) and, although the morphology resembled that of more mature neurons, they failed to express mature neuronal markers.

This constraint to achieve the direct conversion of somatic cells to neurons only with pharmacological tools has already been highlighted by Vasan et al. (2021) and Wang et al. (2022), who note the challenge to seek an optimal formula to achieve acceptable conversion efficiency: not only the optimal concentration of each small molecule in the cocktails is difficult to determine but also the length of the induction time is a concern, as longer times can have toxic effects on the cells, while shorter times might not be effective in inducing neuronal conversion.

In summary, after screening different combinations of small molecules in different culture conditions, only partial reprogramming of the neuro-regenerative OEG-ROB somatic cell line to neuronal-like cells was achieved. From these results we conclude that direct reprogramming of adult olfactory ensheathing glia to mature and functional neurons cannot be induced only with pharmacological tools. This challenge can only be solved through further mechanistic investigation of the reprogramming process by chemical compounds, which could lead to develop a therapy based on OEG induced neuron transplantation in CNS injury or disease.

## Data availability statement

The original contributions presented in the study are included in the article/Supplementary material, further inquiries can be directed to the corresponding authors.

## Ethics statement

The animal study was reviewed and approved by Comité ético de Investigación, Facultad de Ciencias Experimentales, Universidad Francisco de Vitoria.

## Author contributions

MP-L performed experiments and data analysis. DS provided assistance in experimental and data analysis. DF performed the electrophysiological analyses. JS and MTM-F conceived and supervised the project. JS wrote the manuscript. MTM-F, MP-L, DS, and DF contributed to manuscript preparation. All authors contributed to the article and approved the submitted version.
