# Supplementary figures and images for: Small molecules fail to induce direct reprogramming of adult rat olfactory ensheathing glia to mature neurons
Source: Front Mol Neurosci. 2023 Feb 24;16:1110356. doi: 10.3389/fnmol.2023.1110356 (PMC9998535; doi:10.3389/fnmol.2023.1110356)

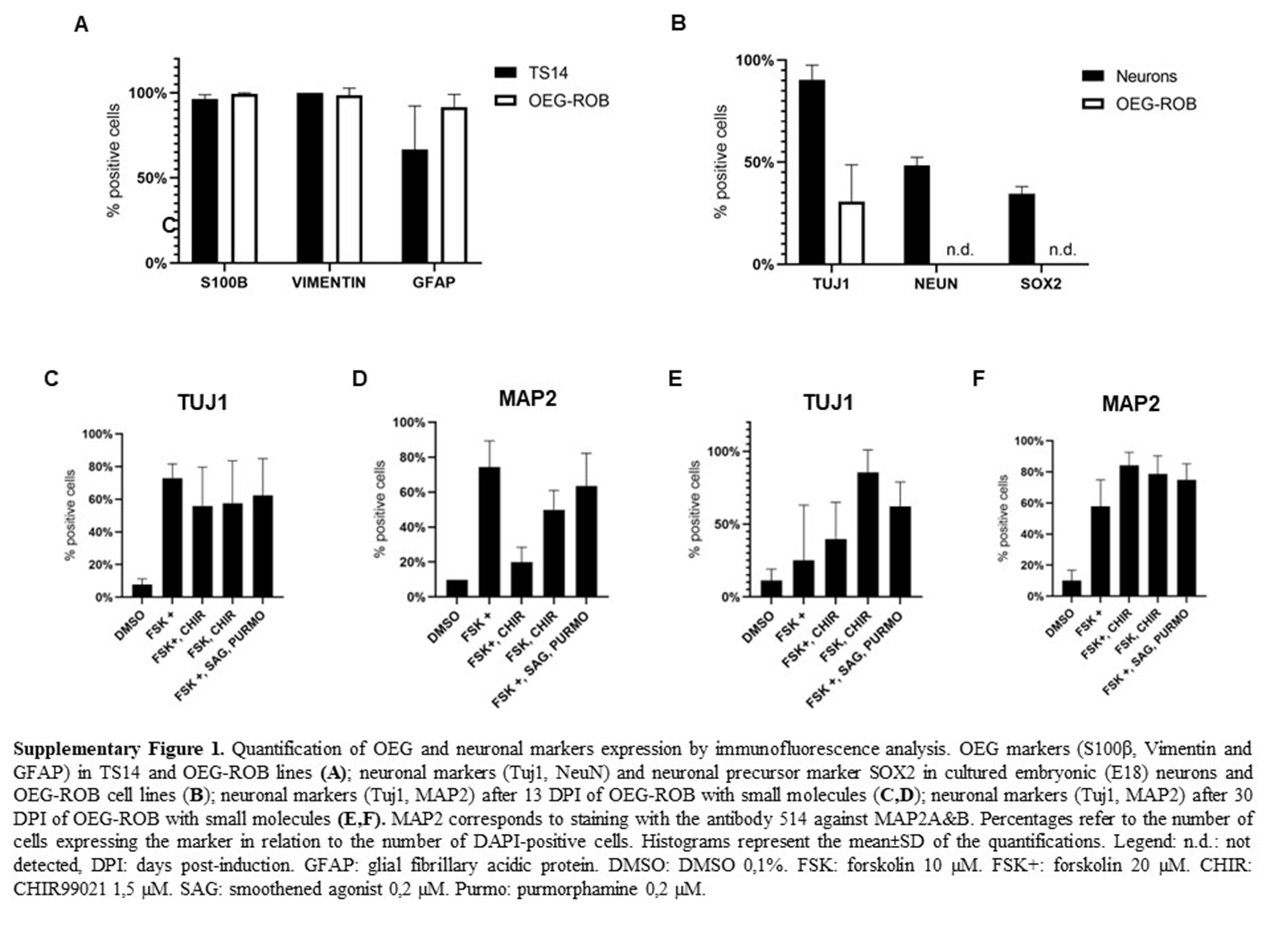

Supplement: Supplementary file 1 [file Image_1.jpg]

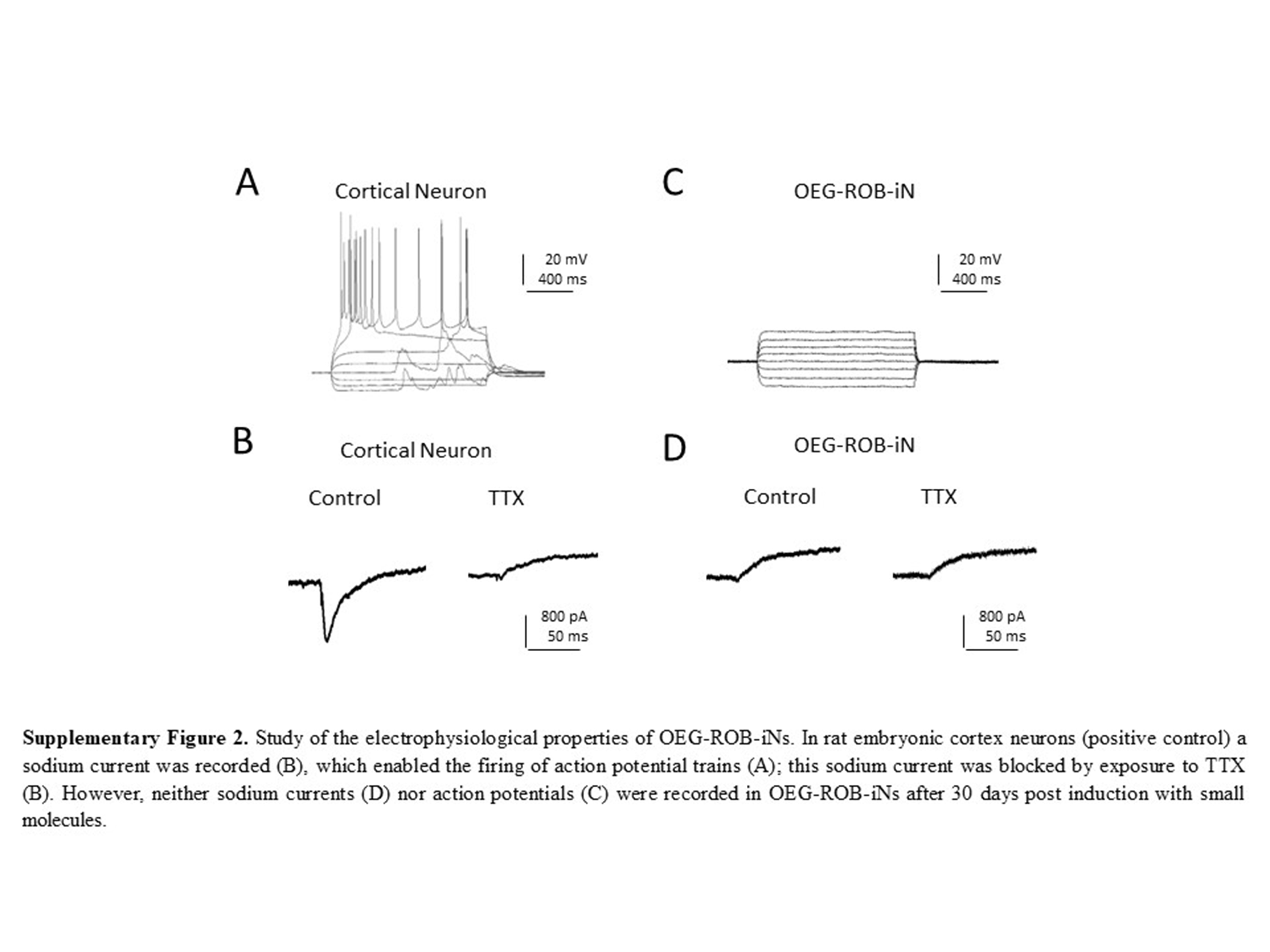

Supplement: Supplementary file 2 [file Image_2.jpg]

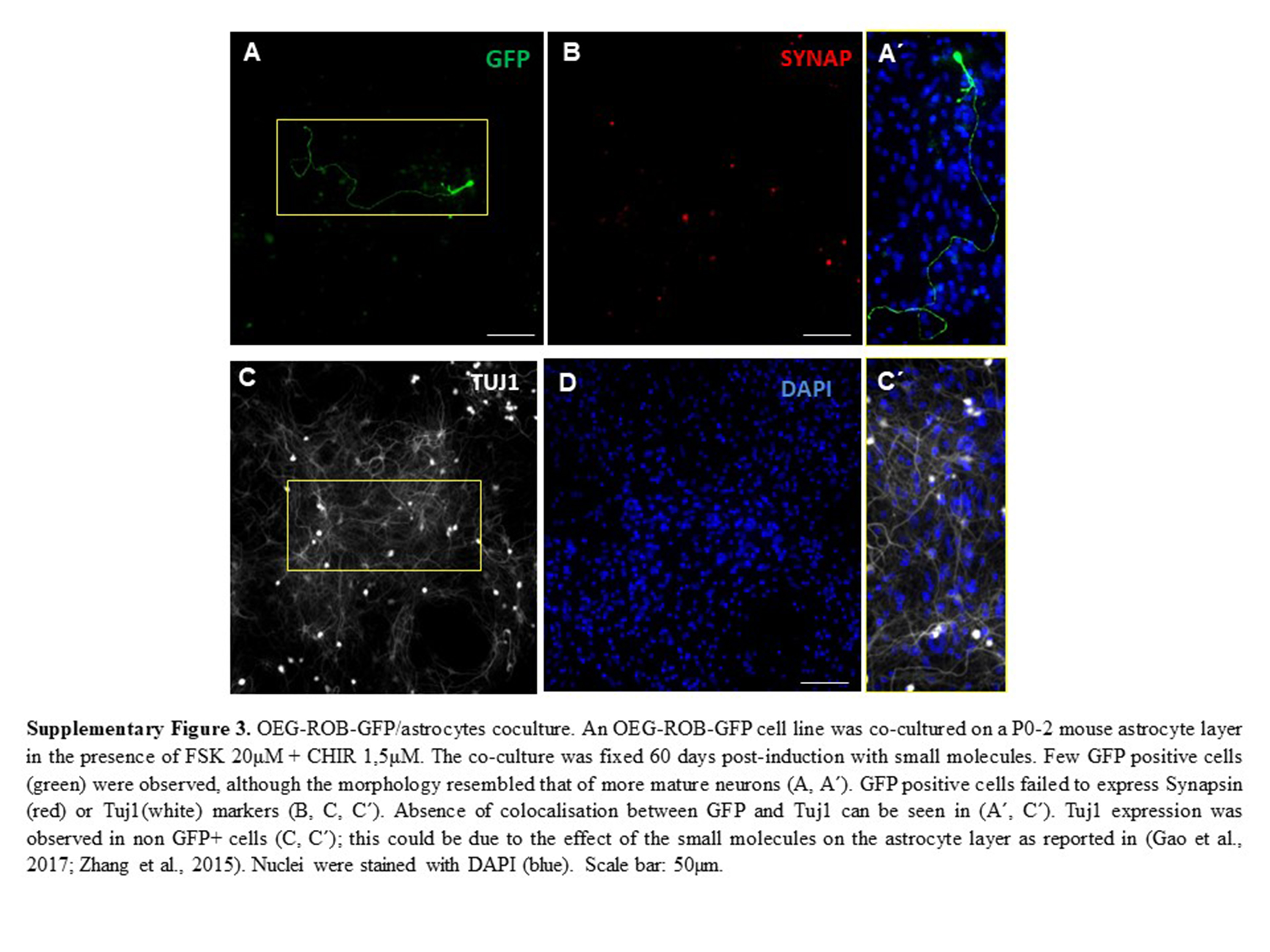

Supplement: Supplementary file 3 [file Image_3.jpg]
