# Supplementary material for: Small molecules fail to induce direct reprogramming of adult rat olfactory ensheathing glia to mature neurons
Source: Front Mol Neurosci. 2023 Feb 24;16:1110356. doi: 10.3389/fnmol.2023.1110356 (PMC9998535; doi:10.3389/fnmol.2023.1110356)
Supplement: Supplementary file 5 [file Table_2.docx]

| ANTIBODY | REACTIVITY* | SOURCE | REFERENCE | RRID | DILUTION |
| --- | --- | --- | --- | --- | --- |
|  |  |  |  |  |  |
| 514 | *PC-Rabbit* | Gift from Dr. Jesús Ávila | *-* |  | 1:500 |
| GFAP | *PC-Guinea Pig* | Synaptic Systems | 173002 | AB_887720 | 1:1000 |
| NeuN | *MC-Rabbit* | Abcam | ab177487 | AB_2532109 | 1:500 |
| S100β | *MC-Mouse* | Sigma | SAB4200671 |  | 1:500 |
| SMI31 | *MC-Mouse* | BioLegend | 801601 | AB_2564641 | 1:500 |
| SOX2 | *PC-Rabbit* | Abcam | ab97959 | AB_2341193 | 1:500 |
| Tuj1 | *MC-Mouse* | Covance | MMS-435P | AB_2315514 | 1:500 |
| Vimentin | *PC-Chicken* | Abcam | ab24525 | AB_778824 | 1:1000 |

*: PC, polyclonal; MC, monoclonal

| ANTIBODY | REACTIVITY | SOURCE | REFERENCE | RRID | DILUTION |
| --- | --- | --- | --- | --- | --- |
|  |  |  |  |  |  |
| 488 | *Chicken* | Thermo Fisher | A-11039 | AB_2534096 | 1:500 |
| 488 | *Mouse* | Thermo Fisher | A-21202 | AB_141607 | 1:500 |
| 488 | *Rabbit* | Thermo Fisher | A-21206 | AB_2535792 | 1:500 |
| 555 | *Chicken* | Thermo Fisher | A-21437 | AB_2535858 | 1:500 |
| 555 | *Goat* | Thermo Fisher | A-21432 | AB_2535853 | 1:500 |
| 555 | *Guinea Pig* | Thermo Fisher | A-21435 | AB_2535856 | 1:500 |
| 555 | *Mouse* | Thermo Fisher | A-31570 | AB_2536180 | 1:500 |
| 555 | *Rabbit* | Thermo Fisher | A-31572 | AB_162543 | 1:500 |
| 594 | *Rabbit* | Thermo Fisher | A-21207 | AB_141637 | 1:500 |
| 647 | *Chicken* | Thermo Fisher | A-21449 | AB_2535866 | 1:500 |
| 647 | *Goat* | Thermo Fisher | A-21447 | AB_2535864 | 1:500 |
| 647 | *Guinea Pig* | Thermo Fisher | A-21450 | AB_2735091 | 1:500 |
| 647 | *Mouse* | Thermo Fisher | A-31571 | AB_162542 | 1:500 |
| 647 | *Rabbit* | Thermo Fisher | A-31573 | AB_2536183 | 1:500 |
